# The Synthesis of Associative Copolymers with Both Amphoteric and Hydrophobic Groups and the Effect of the Degree of Association on the Instability of Emulsions

**DOI:** 10.3390/polym13224041

**Published:** 2021-11-22

**Authors:** Xiaotong Zhang, Gen Li, Yuhao Chen, Keliang Wang, Erlong Yang

**Affiliations:** 1 Department of Petroleum Engineering, Northeast Petroleum University, Daqing 163318, China; zhangxiaotongs@126.com (X.Z.); lg116053717@163.com (G.L.); wkl626@163.com (K.W.); 2 Sinopec Research Institute of Petroleum Engineering, Shelfoil Petroleum Equipment & Services Co., Ltd., Sinopec Group, Dezhou 253034, China; chenyuhao324@126.com

**Keywords:** copolymer, associative, emulsion instability, multiple light scattering

## Abstract

The acrylamide (AM)/methacryloyl ethyl sulfobetaine (SPE)/behenyl polyoxyethylene ether methacrylate (BEM) terpolymer (PASB) was synthesized by soap-free emulsion polymerization. Four types of PASBs were synthesized by adjusting the moles of AM and BEM with constant total moles of monomers. The synthesized copolymers were characterized by Fourier-transform infrared spectroscopy, thermogravimetry, molecular weight, and viscosity. By measuring the microscopic morphology and backscattered light intensity of the emulsions, the instability process of the emulsions prepared by PASBs was investigated in detail. The main instability processes of the emulsions prepared from PASBs within 45 min were flocculation and coalescence. The intermolecular association of copolymer PASBs was dominated by the behenyl functional groups on the molecular chains. The stability of the emulsions, which were prepared from isoviscosity aqueous solutions controlled by the concentration of the associative copolymers, was increased with the degree of association of copolymers. The hydrophobic association between the copolymer molecules can further slow down the flocculation and coalescence of the emulsion droplets on the basis of the same aqueous solution viscosity, which is one of the reasons for improving the stability of the emulsion.

## 1. Introduction

Acrylamide is the most commonly used water-soluble monomer, which can easily synthesize large-molecular-weight polymers. Refs. [1,2] Partially hydrolyzed polyacrylamide (HPAM) [3] is an excellent viscosity-increasing acrylamide polymerization product, which is widely used in enhanced oil recovery [4,5,6,7], drilling fluid systems [8], water treatment [9], biomedicine [10], surface coatings [11], and other fields. In the process of enhanced oil recovery, HPAM is mainly used to increase the viscosity of the salt-containing water phase to control the mobility ratio. The carboxylic acid group in the HPAM structure increases the hydrodynamic volume of the polymer through the Coulomb repulsion in low-salinity water and thus effectively increases the apparent viscosity [12]. However, as the salinity of the formation water of the oil field increases, the viscosity-increasing ability of HPAM is limited [13]. In order to improve the salt tolerance of HPAM, researchers have mainly conducted improvements in two aspects. On the one hand, research is devoted to the synthesis of partially hydrolyzed polyacrylamide with ultra-high molecular weight or rigid groups [14], which can increase the hydrodynamic volume of a single molecular chain. On the other hand, partially hydrolyzed polyacrylamide is modified with associative groups [3], such as the introduction of hydrophobic monomers or new ionic groups.

Amphoteric polymers and hydrophobically associating polymers have special rheological and solution properties in aqueous solutions. Ref. [15] Amphoteric polymers [16] can generate intermolecular associations by the electrostatic attraction of zwitterionic groups in aqueous solutions with high salinity. Moreover, the intermolecular association of hydrophobically associating polymers [17] relies on the spontaneous aggregation of hydrophobic groups. These intermolecular associations all increase the molecular hydrodynamic volume of the polymer and increase the apparent viscosity of the solution.

The methods for synthesizing polymers containing hydrophobic associating groups are mostly inverse emulsion polymerization [18] and micellar copolymerization [19]; however, both methods have some shortcomings [20]. Surfactants, which are not expected to appear in the final products, need to be added during these two syntheses to form micelles or emulsions. In order to remove these surfactants, the product needs to be tediously purified after the copolymerization reaction. However, additional surfactants are not required in the process of soap-free emulsion polymerization [21,22], which simplifies the purification process of the copolymerized product, resulting in a reduction in production costs. Benzoyl polyoxyethylene ether methacrylate (BEM), as a kind of polymerizable surfactant [23,24], can prepare soap-free emulsions. Moreover, the longer behenyl chain in the BEM structure can be used to form a hydrophobic association, so that the aqueous solution has good salt-resistance and thickening performance. Therefore, the surface activity of BEM and the enhancement of polymer association can be used, and monomers with anionic and cationic groups can be added at the same time to synthesize a water-soluble polymer with two associative groups.

Emulsion stability is an indispensable factor in studies related to emulsions since common emulsions are inherently unstable and tend to revert to the stable state of the phases comprising the emulsion [25,26,27]. However, the kinetic stability of emulsions can be increased by emulsifying agents, such as surfactants, nanoparticles, and especially copolymers [28,29,30]. Some studies focused on the effect of the apparent viscosity of copolymers on the stability of emulsions [31,32]. However, this study pays more attention to the effect of copolymers association on droplets in addition to apparent viscosity.

In this work, the soap-free emulsion polymerization method was used to combine acrylamide (AM), amphoteric monomer N-(3-Sulfopropyl)-N-(methacryloxyethyl)-N, N-dimethylammonium betaine (SPE), and surface-active behenyl polyoxyethylene ether methacrylate (BEM) as the raw material to prepare a series of water-soluble polymers with zwitterionic groups and hydrophobic groups. A series of copolymers was used to prepare emulsions. The effect of the ratio of BEM and SPE in the synthesis of copolymers on the instability of the emulsion was further studied.

## 2. Materials and Methods

### 2.1. Materials

Acrylamide (AM), n-(3-sulfopropyl)-n-methacryloxyethyl-n, n-dimethylammoniumbetaine (SPE), and poly(ethylene glycol) behenyl ether methacrylate (BEM) were purchased from Aldrich. Potassium persulfate (KPS), sodium bisulfite (NaHSO_3_), and azobisisobutamidine hydrochloride (AIBA) were purchased from Sinopharm Chemical Reagent Company. All products were used as received. The crude oil and formation water used in viscosity and emulsification measurements were provided by X oilfield. The crude oil had water content of less than 0.5 wt%, and viscosity and acid number of 3.4 mPa·s and 0.031 mgKOH/g (at 56 °C), respectively. The main components of X oilfield injection water are shown in Table 1.

### 2.2. PASB Copolymer Synthesis Steps

In a three-necked flask equipped with reflux condensation, dropping funnel, and N_2_ tube, AM, SPE, and BEM were weighed and poured into deionized water with a certain feeding ratio to form a suspension. Then, N_2_ was purged in the three-necked flask to remove air. Corresponding moles of initiator were weighed and dissolved in water, and slowly dripped into the reaction flask from the dropping funnel. The reaction was first stirred at 20 °C for 2 h, and then the temperature was raised to 45 °C and stirred for 5 h. After the reaction was completed, a milky white viscous liquid product was obtained.

**Figure 1:**
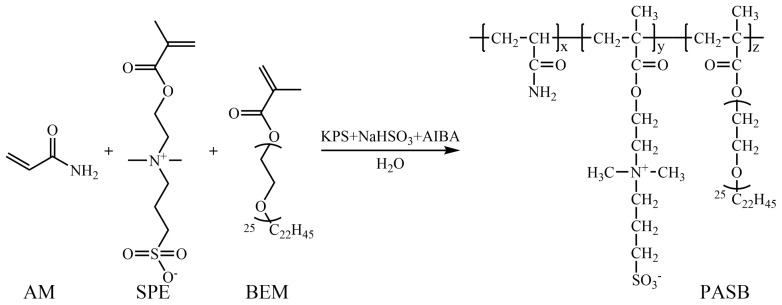
Synthetic route of PASB copolymers.

After stopping the reaction, the copolymer was precipitated and washed with acetone. The hardened product, after purification, was ground and dried in a vacuum drying oven. Finally, a white powdery polyacrylamide/methacryloyl ethyl sulfobetaine/behenyl polyoxyethylene ether methacrylate terpolymer (PASB) was obtained. The synthesis reaction formula of PASB is shown in Figure 1.

The ratio of reactive monomers and initiators is listed in Table 2. The concentration of the reactive monomers was constant at 1 mol/L.

### 2.3. FTIR, TGA, and DTG Measurement

FTIR spectra were taken with a 1615 FTIR (PerkinElmer, Waltham, MA, USA) spectrometer using KBr pellet pressing method for all PASBs. A Q5000IR thermogravimetric analyzer (TA, New Castle, DE, USA) was used to perform thermogravimetric analysis (TGA) and differential thermogravimetric analysis (DTG) on samples. The programmed heating rate was 10 °C/min.

### 2.4. PASB Average Molecular Weight

The average molecular weight of PASBs was measured using a BI-200SM wide-angle dynamic/static laser-scattering instrument (Brookhaven, Holtsville, NY, USA). The measurement was carried out in a 0.10 mol·L^−1^ NaCl aqueous solution to shield the charge on copolymers. The concentration of the copolymers was 1 × 10^−5^ g·mL^−1^.

### 2.5. Viscosity Measurement of PASB

The apparent viscosity of PASB solutions was measured using a DV-III+ viscometer (Brookfield, New York, NY, USA) at 56 °C with a constant shear rate of 7.34 s^−1^.

### 2.6. Preparation of Aqueous Phases and Emulsion

Before use, the crude oil was stored in a refrigerator protected by argon to prevent oxidation. Prior to emulsification, the oil was heated to 56 °C and shaken to ensure a homogeneous mixture for sampling. The four types of PASBs were separately dissolved in oilfield water to prepare four kinds of aqueous solutions as the emulsified water phases. Different concentrations of copolymers were used in four kinds of PASB solutions in order to ensure that the viscosities of the solutions were close to 25 mPa·s. Emulsions were made from a volume of oil and a known composition of PASB solution, constantly 3/2 (*v*/*v*); a total of 50 mL placed in a 100 mL beaker, with an IKA T25 ultra turrax homogenizer (Germany) at 4500 rpm for 10 min in a 56 °C water bath.

### 2.7. Optical Microscopic Observation

The emulsion type and drop size were examined by optical microscopy techniques. For optical microscopy, small samples of the emulsion were placed in a single cavity cell and observed with an Olympus IX73 microscope fitted with a sCMOS digital camera. Small samples of the emulsion were taken from the middle of the emulsion using a plastic dropper immediately after the emulsion was formed. The single cavity cell was used in order to keep the droplets from being blocked by each other as much as possible when viewed. The same light intensity was used for all observations.

### 2.8. Multiple Light-Scattering Measurements

The stability of crude oil emulsion stabilized by PASB solutions was monitored by measuring the backscattering and transmission of a pulsed near-infrared light (880 nm) by Turbiscan Lab Expert (Formulaction, Toulouse, France). The transmittance detector received the light which passed through the dispersion at an angle of 180° with respect to the source, while the backscattering detector received the light scattered backward by the dispersion at an angle of 45°.

There are several types of emulsion instability phenomena, such as the flocculation and coalescence between droplets, as well as the creaming and sedimentation of droplets which lead to phase separation. Using Turbiscan Lab Expert to analyze the light intensity in the middle part of the backscattered spot formed after the light passed through the opaque emulsion sample, stability changes at different times and locations within the emulsion system can be studied. The relationship between the backscattered light intensity and the photon transfer mean free path of photons in the dispersion system is as follows [33,34]:

(1) $$BS=\frac{1}{\sqrt{l^{*}}}$$

(2) $$l^{*}(d,\varphi )=\frac{2d}{3\varphi (1-g)Q_{s}}$$

where *BS* is the flux (intensity) of backscattered light, l* is the mean free path of photon motion, d is the particle size of the dispersed phase droplet, *φ* is the volume fraction of the dispersed phase, and *g*, *Q_s_* are optical parameters given by Mie theory (cflight scattering). From Equations (1) and (2), the backscattered light intensity (*BS*) is proportional to the dispersed phase volume fraction (*φ*) and inversely proportional to the dispersed phase droplet size (*d*).

Immediately after the emulsion was formed, the measuring cell was filled with 20 mL of sample to be analyzed at a temperature of 56 °C. The entire height of the various samples was scanned for 45 min. In this study, the delta backscattering was selected as the basis for further analysis since the transmitted light value of the emulsion containing the crude oil was less than 0.2%. The experiment was repeated three times under the same experimental conditions.

## 3. Results and Discussion

### 3.1. FTIR of Copolymer PASB

**Figure 2:**
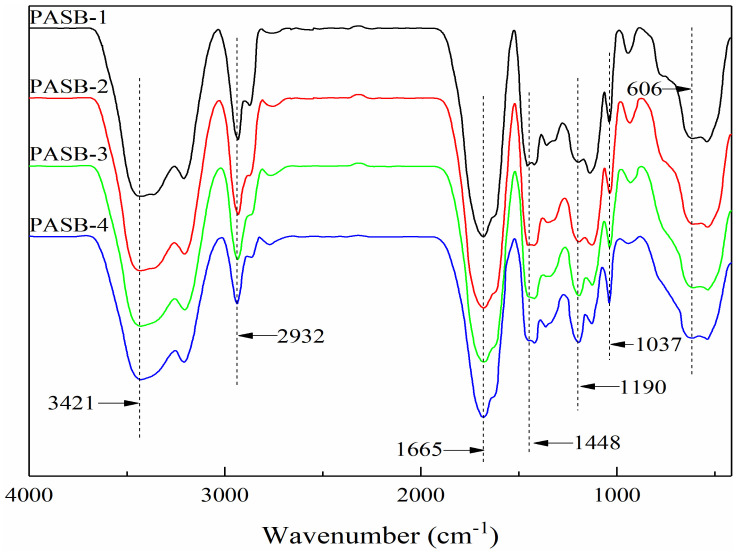
Fourier transform infrared spectra of copolymers PASBs.

The Fourier transform infrared spectrum of a series of PASB copolymers is shown in Figure 2. The absorption peaks at 3421 cm^−1^, 1665 cm^−1^, and 1448 cm^−1^ are the N-H stretching vibration peak, C=O stretching vibration peak, and the mixed in-plane bending vibration peak of C-N and N-H of the amide, respectively. These indicate that the copolymer contains AM. The absorption peaks at 1037 cm^−1^ and 606 cm-1 are the symmetrical vibration absorption peak of -SO_3_^−^ and the stretching vibration absorption peak of C-S, respectively, indicating that the copolymer structure contains the characteristic absorption peak of SPE. The C-O (ether) stretching vibration absorption peak in the BEM structure is 1190 cm^−1^. The stretching and bending vibration peaks of -CH_3_ and -CH_2_ are 2932 cm^−1^, 2774 cm^−1^, and 1365 cm^−1^. It can be seen that four target copolymers, PASBs, have been synthesized. Moreover, it is worth noting that the absorption peak at 1190 cm^−1^ from PASB-1 to PASB-4 gradually becomes larger, which means that the ether bond of BEM is effectively increased in the copolymer PASBs. This indirectly indicates that the behenyl group of BEM in the copolymer PASB has also increased. In addition, the absorption peak at 1448 cm^−1^ from PASB-1 to PASB-4 is slightly weakened, indicating that the content of amino groups in the copolymers is reduced. This proves again that the number of hydrophobic groups of BEM in molecules is gradually increased from PASB-1 to PASB-4, while the number of amino groups of AM in molecules is gradually decreased.

### 3.2. Thermal Properties of Copolymer PASB

**Figure 3:**
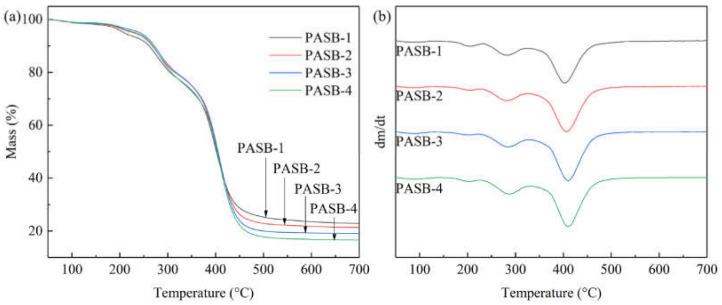
TGA and DTG curves of copolymers PASB. (**a**) TGA. (**b**) DTG.

The TGA and DTG curves of PASBs are shown in Figure 3. According to the measurement results in Figure 3, it is found that the thermal weight loss diagram of PASB polymers with different BEM content is similar to the decomposition temperature, but the weight loss percentage changes. Before 100 °C, the loss of mass is the evaporation of water absorbed by the polymer. Importantly, decomposition of polymer PASB is mainly carried out through the following three steps: the first step is around 230 °C, which may be the decomposition of amide bonds. Moreover, the decomposition temperature of polymer amide bond decreases with the increase in BEM content, which may be due to the huge side group of BEM destroying the hydrogen bond between amide groups. The second step is around 310 °C, which may be because SPE loses weight and decomposes at this temperature. The third step mainly occurs at 410 °C, which may be due to the degradation of the polymer backbone and BEM side groups. In addition, as the content of hydrophobic monomer BEM increases, the decomposition temperature of the polymer backbone and BEM side groups increases (Figure 3b), indicating that the temperature resistance of the polymer increases.

### 3.3. Molecular Weight of Copolymer PASB

In static light-scattering, the apparent weight average molecular weight (Mw. app) and the second virial coefficient (A2) of the polymer can be accurately obtained by measuring the dependence of the time-average scattered light intensity on the angle and concentration. The Mw and A2 of PASB in NaCl aqueous solution, as shown in Table 3, were obtained by post-processing of the instrument software. The molecular weight of the polymers PASB-1 to PASB-4 gradually decreases, which may be affected by the increase in the viscosity of the solution systems. The A2 values of this series of polymers were all positive, indicating that the four copolymers PASB were well dissolved in the 0.10 mol·L^−1^ NaCl aqueous solution.

### 3.4. Influence of PASB Copolymer Concentration on Solution Viscosity

**Figure 4:**
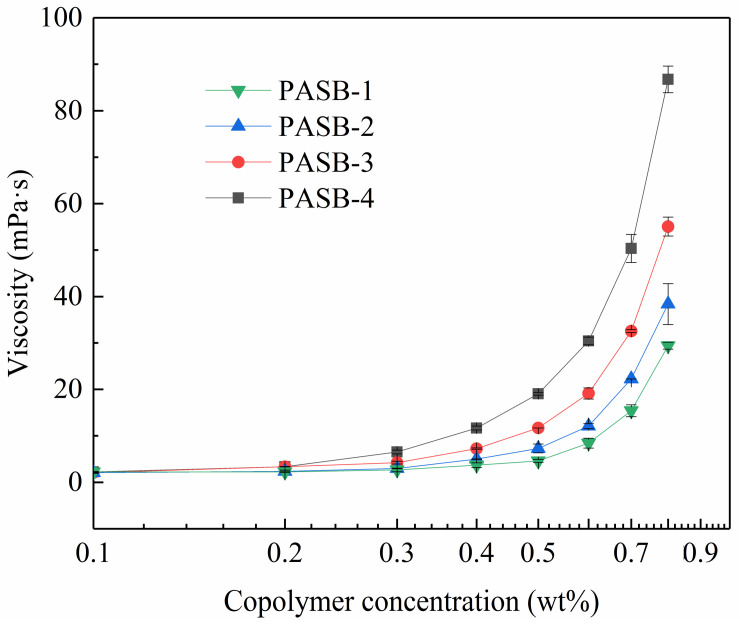
Viscosity of PASB copolymers as a function of copolymer concentrations at 56 °C.

It is very necessary to study the viscosity of associative polymers. The multiple molecular chains of copolymers are interwoven with each other through hydrophobic association to form a three-dimensional network (physical cross-linked structure), which significantly increases the hydrodynamic volume and apparent viscosity. The apparent viscosity of the four copolymers of PASB in the formation water is shown in Figure 4. At 56 °C, the apparent viscosity of all PASBs solutions increased with the concentration. All viscosity curves of PASBs clearly show two different regimes divided by critical association concentration [15]: a first unentangled or intramolecular association regime where the viscosity increases moderately, and a second intermolecular association regime where the viscosity varies according to a power law [35]. To illustrate this variety, the viscosity curve of PASB-1 was analyzed as an example. When the concentration of PASB-1 is less than 0.5 wt%, the viscosity change is not obvious. The reason for this phenomenon is that the probability of hydrophobic groups contacting and associating between molecules to increase viscosity is low in the diluted polymer solution. When the concentration of the polymer solution was increased to more than 0.5 wt%, the possibility of intermolecular contact and association was also increased, which is manifested as a rapid exponential increase in viscosity. As the ratio of behenyl functional groups in the molecular chain from PASB-1 to PASB-4 decreased, the critical association concentration was detected to decrease sequentially, indicating that the behenyl group in the molecular chain plays a leading role in the association of PASB copolymers.

### 3.5. Emulsion Microscopic Morphology

**Figure 5:**
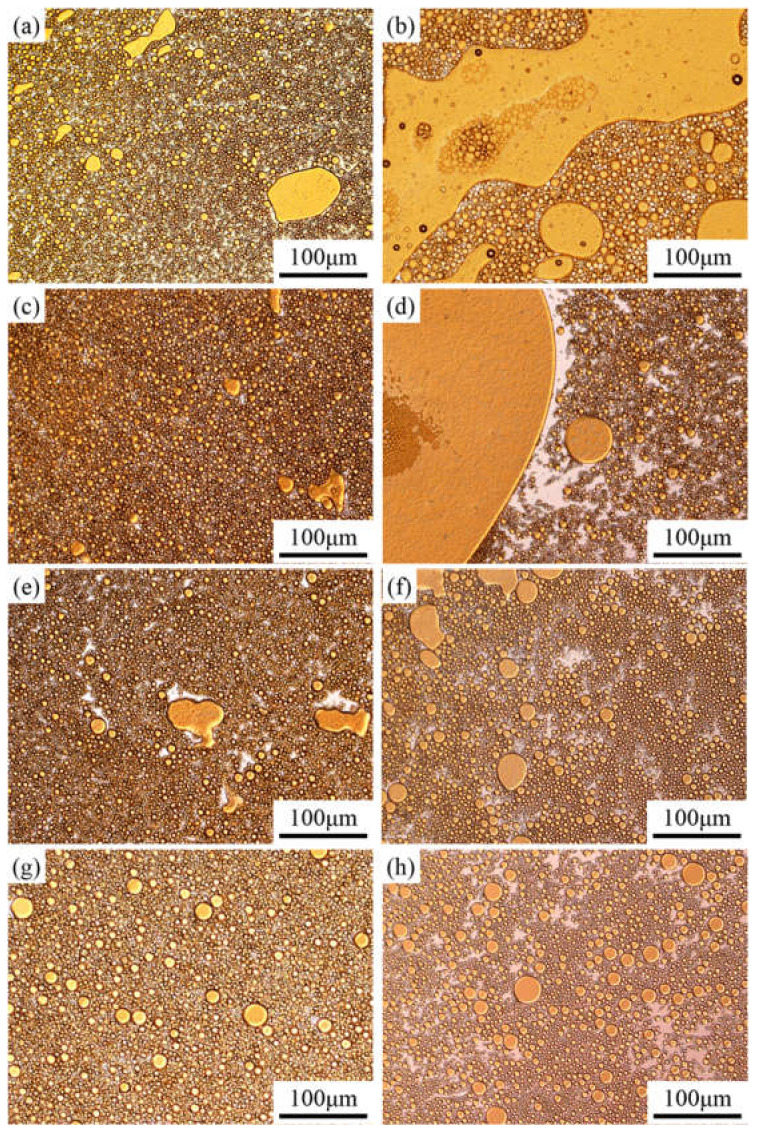
Optical micrographs of emulsions prepared from four kinds of PASBs with the same aqueous solution viscosity. Partial yellow is oil, and white is water. (**a**) Fresh, prepared from PASB-1; (**b**) 45 min later, prepared from PASB-1; (**c**) fresh, prepared from PASB-2; (**d**) 45 min later, prepared from PASB-2; (**e**) fresh, prepared from PASB-3; (**f**) 45 min later, prepared from PASB-3; (**g**) fresh, prepared from PASB-4; (**h**) 45 min later, prepared from PASB-4.

Before studying the process of emulsion instability, it is important to understand the emulsion type and particle size. The optical micrographs of the emulsions stabilized by the PASB solutions with constant viscosity (25 mPa·s) are shown in Figure 5.

According to the microscope observation results (Figure 5a,c,e,g), the emulsions containing the four PASB polymers are all O/W types in the initial state, and the particle size distribution is relatively uniform.

After standing for 45 min, the type of emulsion in the middle of the bottle still maintains O/W, but the particle size changes significantly. At the sampling point (the middle position of the bottle), the particle size and oil content of the emulsion stabilized by PASB-1 and PASB-2 after standing for 45 min (Figure 5a,c) were greater than the initial state (Figure 5b,d). In Figure 5b,d, it is easy to observe a continuous, larger area of the oil phase. This is caused by the continuous coalescence and creaming of oil droplets. However, in the emulsion stabilized by PASB-3 and PASB-4, the difference between the fresh emulsions (Figure 5e,g) and the emulsions after standing for 45 min (Figure 5f,h) is not significant. Even though the original aqueous solutions of the four emulsions prepared by PASB have the same viscosity, the microscope images preliminarily show that the stability of the emulsion is affected by the molecular structure of the copolymers.

### 3.6. Changes in the Backscattered Light Intensity of the Emulsions

**Figure 6:**
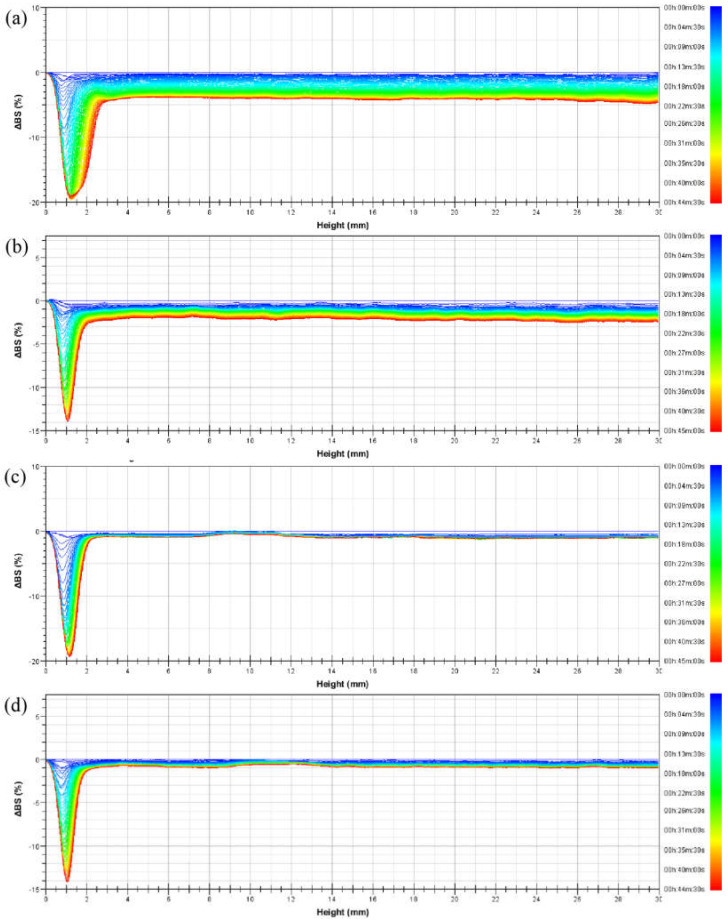
Delta backscattered light intensity profiles of emulsions prepared from (**a**) PASB-1, (**b**) PASB-2, (**c**) PASB-3, and (**d**) PASB-4, respectively.

In order to study the dynamic process of emulsion instability, multiple light-scattering tests were performed on the emulsions, which were allowed to stand at 56 °C. The delta backscattered light intensity (Δ*BS*) of emulsions prepared from four kinds of PASB systems within 45 min is shown in Figure 6.

According to Figure 6, the Δ*BS* of the four emulsions at the bottom of the measuring cell decreased relatively significantly with time. The main reason for the decrease in light intensity here is the decrease in the oil phase volume fraction (φ) caused by the rise of oil droplets. After the droplets of larger particle size leave together from the bottom of the measuring cell, the remaining oil volume fraction is smaller than the upper part. This causes a sudden change in Δ*BS* between the bottom and the top. As time increases, the minimum value of Δ*BS* continues to decrease and moves to the left, which represents a phase separation at the bottom of the measuring cell. Such phase separation was measured when the emulsion stabilized by PASB-1 occurred in the measuring cell height range of 0 mm to 3 mm, while the emulsions stabilized by PASB-2, PASB-3, and PASB-4 occurred in the range of 0 mm to 2 mm. This means that the emulsion stabilized by PASB-1 is easier to phase-separate than the other three emulsions.

Compared with the 30 mm height emulsion in the measuring cell, the degree of phase separation at the bottom of the measuring cell was not obvious; thus, the influence on the oil–water ratio in the upper part of the measuring cell was limited. In the range of 3 mm to 30 mm in the measuring cell, the lines of Δ*BS* decreased relatively parallel. The oil phase volume fraction (φ) of this part did not change significantly. According to Equations (1) and (2), increase in droplet diameter (d) leads to a decrease in *BS*. This indicates that the main types of instability of the emulsion in the height range of 3 mm to 30 mm are flocculation and coalescence. From Figure 6a–d, the degree of this flocculation and coalescence tends to decrease, indicating that the increase in the proportion of the BEM monomer in the molecular chain effectively improves the stability of the emulsion from flocculation and coalescence.

### 3.7. The Relationship between TSI and Reactive Monomers Ratio

Emulsion stability can also be characterized using the Turbiscan Stability Index (*TSI*) [33,34]. The *TSI* value is calculated as follows:

(3) $$TSI=\sum_{i}\frac{\sum_{h}\left|scan_{i}(h)-scan_{i-1}(h\left.)\right|\right.}{H}$$

where *scan_i_(h)* is the light intensity of the *i*-th *scan* at a height of *h*, and H is the total height of the measured sample.

**Figure 7:**
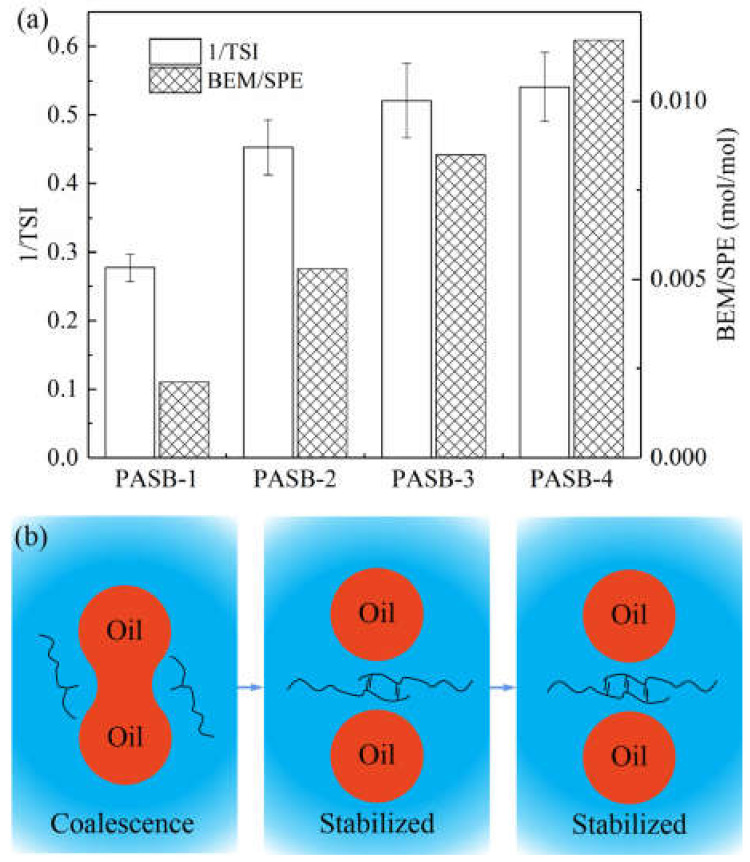
The relationship between emulsion stability and polymer intermolecular association. (**a**) Correspondence between 1/*TSI* and BEM/SPE ratio. (**b**) A schematic diagram of the intermolecular association of polymers preventing the coalescence of oil droplets. When PASBs have effectively prevented the occurrence of flocculation and coalescence, the stability is difficult to be further improved.

The comprehensive influence of multiple unstable phenomena on the change of light intensity can be characterized by the reciprocal of *TSI*. The larger the 1/*TSI* value, the more stable the system. The molar ratio of BEM to SPE monomer in the synthesis of PASBs and the 1/*TSI* of the corresponding emulsions after standing for 45 min are shown in Figure 7a.

It can be seen from Figure 7a that the emulsion stability and the ratio of BEM/SPE present a positive correlation. However, as the ratio of BEM/SPE continues to increase, the increasing trend of emulsion stability slows down and reaches its limit. The principle of this emulsion stabilization process is illustrated in Figure 7b. Under the condition of the same aqueous solution viscosity, the increase in the content of behenyl groups in the PASB molecular chain can significantly increase the degree of hydrophobic association of the PASB polymer, effectively preventing the flocculation and coalescence of the emulsion. However, this improvement has limits. When the flocculation and coalescence of the emulsion within the measurement time range are quite insignificant, continuously increasing the ratio of BEM/SPE can cause the emulsion to maintain its initial state during the standing process, but will not continue to significantly increase the emulsion stability.

## 4. Conclusions

(1) The soap-free emulsion polymerization method was used to prepare a variety of water-soluble copolymers with both ultra-long hydrophobic chains and amphoteric groups. Through the action of a mixed initiator composed of KPS/NaHSO_3_/AIBA, different proportions of AM, SPE, and BEM were copolymerized to obtain four PASB copolymers.
(2) The main instability processes of the emulsions prepared from PASB in 45 min were flocculation and coalescence. The phase-separation phenomenon was not significant in PASB-1 to PASB-4 emulsions. The type of all emulsions during the standing for 45 min was always O/W.
(3) The behenyl functional group in the molecular chain of the synthesized PASB copolymer plays a leading role in the intermolecular association. Increasing the ratio of BEM/SPE significantly increased the degree of hydrophobic association of PASB polymers, which can cause the emulsion to reduce the occurrence of flocculation and coalescence during the standing process, but will not continue to significantly improve the stability of the emulsion, although the viscosity of the aqueous solution in the emulsion was controlled to be always equal.
(4) The stability of the emulsions, which were prepared from isoviscosity aqueous solutions controlled by the concentration of the associative copolymers, was increased with the degree of association of copolymers.

## Figures and Tables

**Table 1:** Ionic composition of X oilfield water.

Items	Value
K^+^ (mg/L)	18.7
Na^+^ (mg/L)	3907.3
Ca^2+^ (mg/L)	41.4
Mg^2+^ (mg/L)	118.4
Cl^−^ (mg/L)	4230.7
SO_4_^2−^ (mg/L)	138.0
HCO^3−^ (mg/L)	3675.2
Total salinity (mg/L)	12129.7

**Table 2:** The feeding composition of copolymers PASB.

Sample	AM (mol%)	SPE (mol%)	BEM (mol%)	KPS (mmol)	NaHSO_3_ (mmol)	AIBA (mmol)	Yield (%)
PASB-1	94.8	5	0.2	0.022	0.045	0.133	93.06
PASB-2	94.5	5	0.5	0.022	0.045	0.133	92.73
PASB-3	94.2	5	0.8	0.022	0.045	0.133	89.20
PASB-4	93.9	5	1.1	0.022	0.045	0.133	87.27

**Table 3:** Static laser-light-scattering data for PASBs.

Sample	Mw. App (g × mol^−1^)	A2 (10^−5^ mol × mL × g^−2^)
PASB-1	(1.134 ± 0.093) × 10^6^	6.458 ± 0.967
PASB-2	(9.703 ± 0.517) × 10^5^	5.681 ± 0.881
PASB-3	(8.073 ± 0.355) × 10^5^	3.861 ± 1.019
PASB-4	(5.981 ± 0.191) × 10^5^	4.269 ± 0.949
